# Tryptophan Metabolism and COVID-19-Induced Skeletal Muscle Damage: Is ACE2 a Key Regulator?

**DOI:** 10.3389/fnut.2022.868845

**Published:** 2022-04-08

**Authors:** Hikari Takeshita, Koichi Yamamoto

**Affiliations:** Department of Geriatric and General Medicine, Osaka University Graduate School of Medicine, Osaka, Japan

**Keywords:** angiotensin converting enzyme 2 (ACE2), tryptophan, skeletal muscle, COVID-19, SARS-CoV2

## Abstract

The severity of coronavirus disease 2019 (COVID-19) is characterized by systemic damage to organs, including skeletal muscle, due to excessive secretion of inflammatory cytokines. Clinical studies have suggested that the kynurenine pathway of tryptophan metabolism is selectively enhanced in patients with severe COVID-19. In addition to acting as a receptor for severe acute respiratory syndrome coronavirus 2, the causative virus of COVID-19, angiotensin converting enzyme 2 (ACE2) contributes to tryptophan absorption and inhibition of the renin-angiotensin system. In this article, we review previous studies to assess the potential for a link between tryptophan metabolism, ACE2, and skeletal muscle damage in patients with COVID-19.

## Introduction

The global spread of coronavirus disease 2019 (COVID-19) is a serious ongoing issue owing to the high infectiousness of the causative virus, severe acute respiratory syndrome coronavirus 2 (SARS-CoV2), and encroaching resistance to vaccine therapy due to viral mutations. One of the serious challenges of this disease is that some patients who become seriously ill, ultimately develop fatal acute respiratory distress syndrome (ARDS). The prognostic factors are not thoroughly elucidated, but clinical studies have shown that the disease is more severe in men compared to women, specifically in the elderly, and depends on underlying lifestyle-associated chronic diseases, including diabetes and hypertension. Initial symptoms resemble common influenza symptoms. If severe pneumonia develops, symptoms of ARDS can appear, with increased blood levels of pro-inflammatory cytokines including IL-6, IL-10, and TNF-α, and decreased frequency and abnormal functionality of T lymphocytes (1–3). Generation of an excessive inflammatory response, called a cytokine storm, is a probable molecular mechanism underlying the development of ARDS (4–6). In skeletal muscle, myalgia and increased levels of creatine kinase, together with signs of skeletal muscle damage are more pronounced in critically ill patients at higher risk of mortality (7–9). Examination of postmortem skeletal muscle specimens from critically ill patients revealed an inflammatory signature, independent of direct viral effects (10), suggesting that excessive immune activation throughout the body leads to organ damage in severe disease scenarios. It is of considerable interest to note that interferon (IFN) type I reactivity, which is normally elevated upon viral infection in COVID-19 patients, is attenuated with increasing severity, in contrast to IL-6, IL-10, and TNF-α. One possible mechanism could be the presence of proteins with strong IFN1 suppressive activity in SARS-CoV2, including ORF3b and ORF6 (11, 12).

## Changes in Tryptophan Metabolism in Patients With Severe COVID-19

Metabolomics analyses of serum samples from COVID-19 patients have revealed the involvement of metabolites of the kynurenine pathway (responsible for metabolism of the essential amino acid tryptophan) in the immune response to SARS-CoV2 infection (13, 14). Previous studies have shown decreased serum tryptophan levels, and increased levels of intermediate metabolites [kynurenine (KYN) and kynurenic acid (KYNA)] in patients with severe COVID-19 compared to healthy controls (13, 14). These differences in KYN and KYNA levels, as well as the reactivity to these metabolites, have been suggested to be contributing factors to the enhanced severity of COVID-19 in male patients (14). In males, the ratio of KYNA to KYN (KA:K) is elevated in patients with severe disease, and KA:K positively correlates with blood levels of IL-6. In females, KA:K does not correlate with disease severity or particular cytokine levels (14), indicating that biological responses to KYN and KYNA are important in determining the prognosis of COVID-19.

Dietary tryptophan absorption occurs mostly in the small intestine, whereas tryptophan metabolism occurs mainly in the liver and kidneys. Approximately 95% of absorbed tryptophan is metabolized by the kynurenine pathway, and the rate-limiting enzymes are tryptophan-2, 3-dioxygenase (TDO) and indoleamine-2, 3-dioxygenase 1/2 (IDO1/2), which metabolize tryptophan to KYN. Under physiological conditions, TDO, which is mainly expressed in the liver, is regulated by hormones including cortisol, insulin, glucagon, and epinephrine (15), while IDO1, which is expressed in monocytes and dendritic cells in organs other than the liver, is activated by pro-inflammatory cytokines such as IFN-γ, IL-6, and TNF-α. The recently identified IDO2 gene, which displays 45% homology with IDO1, is constitutively expressed in the brain, liver, kidney, and epididymis. However, its tryptophan metabolic activity is thought to be lower than that of IDO1 (16). According to previous studies, blood levels of serotonin, a kynurenine pathway-independent metabolite of tryptophan, were lower in patients with severe COVID-19 compared to healthy controls (13), indicating that tryptophan utilization during SARS-CoV2 infection is specifically facilitated by the kynurenine pathway.

## Immunomodulatory Mechanisms Mediated by AhR Activation by IDO1 and Tryptophan Metabolites

IDO1 and its kynurenine pathway metabolites have been actively studied as immune regulators because of their involvement in fetal-maternal immune tolerance (17). In cancer immunity, IDO1 and TDO, which are highly expressed in various tumors, including colon and gastric cancers, are thought to be involved in the pathogenesis of immune tolerance in cancer cells (18–20). These cells evade the immune system by activating IDO1, which suppresses T cell proliferation by depleting tryptophan in their microenvironment, and suppresses differentiation of naive CD4^+^ T cells into regulatory T (Treg)cells *via* the immunosuppressive properties of tryptophan metabolites (21–26). This immunosuppressive effect of IDO1 has also been observed in pulmonary inflammatory diseases, where its activity increased in response to elevated IFNs in lung epithelial cells during microbial infection. In this regard, the KYN-AhR (aryl hydrocarbon receptor) pathway is thought to contribute to the immunosuppressive function of IDO1 (27). KYN and KYNA are ligands of AhR, a receptor-type transcription factor activated by cyclic aromatic hydrocarbons (28). KYNA, which is generally thought to contribute to immunosuppressive functions by regulating cytokine release from invariant natural killer T cells through G protein-coupled receptor 35 (GPR35) activation (29), has been reported to promote IL-6 secretion in experimental breast cancer cells by binding to AhR (30). Since IL-6 is an immunomodulator involved in both inflammatory and anti-inflammatory functions (31), it remains unclear whether the binding of KYNA to AhR contributes to the promotion or suppression of immune responses. It has been shown that physiological concentrations of KYN promote Treg differentiation from naive T cells in an AhR-dependent manner *in vitro* during inflammation (26). Tregs, including Tr1 cells, CD8^+^ Tregs, and FoxP3^+^ Tregs, are involved in immunosuppression through secretion of TGF-β and IL-10. In contrast, Th17 cells, which differentiate in the presence of TGF-β, IL-6, or IL-21 (31, 32), are known to be involved in tissue inflammation by secreting IL-17A, IL-17F, IL-21, IL-22, and TNF-α. Although Treg and Th17 cells have contradictory functions, they share a requirement for TGF-β for differentiation. AhR agonism may also play a role in promoting differentiation of both cell types (33, 34), where TGF-β stimulation increases AhR expression, which in turn increases ligand-binding activity (26). Thus, Tregs and Th17 cells are known to have plasticity and the ability to display similar traits to each other under the influence of cytokines and other factors (35).

**Figure 1:**
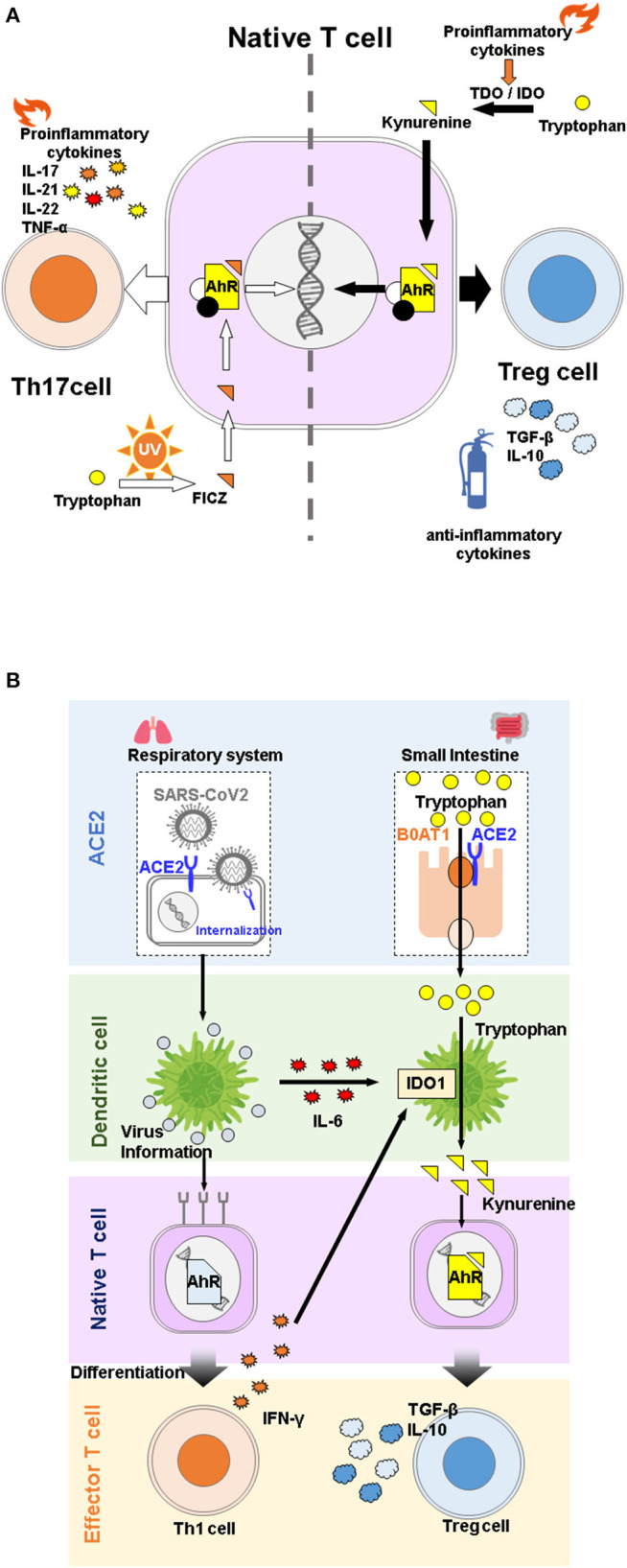
**(A)** Effects of different tryptophan metabolites on differentiation of native T cells *via*. aryl hydrocarbon receptor (AhR) activation. Despite belonging to the same pathway, 6-Formylindolo[3,2-b]carbazole (FICZ) promotes differentiation of native T cells into Th17 cells, and induces inflammation, while kynurenine promotes differentiation into Treg cells and functions as an anti-inflammatory agent. **(B)** Overview of angiotensin converting enzyme 2 (ACE2) physiological functions during COVID-19 infection. SARS-CoV2 infection stimulates secretion of proinflammatory cytokines, which in turn activates IDO1 and promotes the immunosuppressive system through kynurenine binding to AhR. ACE2 is a receptor for SARS-CoV2 but is also involved in immune regulation through tryptophan absorption.

AhR is involved in regulating helper T cell differentiation and macrophage activation, and is therefore involved in both acquired and innate immunity. Its ligands include dioxin and tryptophan metabolites (36). Dioxin is known to promote the generation of Tregs by activating AhR (34). On the other hand, 6-formylindolo[3,2-b]carbazole (FICZ), a compound produced by UV irradiation of tryptophan, and also an AhR agonist and tryptophan metabolite, has been reported to promote the generation of Th17 cells (26, 33). These reports indicate that the immunosuppressive response to AhR agonism can produce different results, depending on the type of ligand (Figure 1A). One possible reason is that the ligands themselves undergo AhR-induced enzymatic modification by cytochrome P450, details of which remain unclear (26).

In the case of innate immunity, increased production of type I IFN in IDO1-deficient mice infected with murine leukemia virus or wild type mice treated with IDO inhibitors (37) and suppression of type I IFN secretion in the presence of AhR *in vitro* (36) suggest that the metabolites produced by IDO1 suppress type I IFN secretion *via* AhR activation, but the ligand specificity is still unclear. Therefore, in addition to the ORF gene sequence in SARS-CoV2, AhR activation by KYN may be involved in type I IFN suppression in patients with severe COVID-19 (38), suggesting that activation of the IDO1-KYN-AhR pathway promotes mucus secretion in alveolar epithelial cells and induces hypoxia in patients with COVID-19 (39). However, during the cytokine storm observed in severe COVID-19, KYN mediated AhR activation is considered to be an essential biological response to suppress systemic organ damage (13, 40), as is mucus secretion in terms of infection defense. Therefore, AhR activation by IDO1-derived tryptophan metabolites in patients with severe COVID-19 can be regarded as a biological defense mechanism against severe infection (13).

Tryptophan metabolites produced by catabolism in gut microbiota are thought to mediate immunomodulatory functions *via* activation of AhR, KYN, and KYNA (41–44). Data from mouse models and patients with Inflammatory Bowel Disease (IBD) suggest that AhR activation exerts a preventive effect on the development of colitis (45–48). A possible mechanism underlying this phenomenon is that AhR activation by tryptophan metabolites promotes the differentiation of native T cells in the intestinal epithelium into CD4^+^CD8α^+^ T-cells, which are responsible for the anti-inflammatory effects complementary to Tregs (46). On the other hand, IL-22, a cytokine secreted by innate lymphoid cells upon infection with pathogenic bacteria enhances defense against bacterial infection by producing mucus and antimicrobial proteins in intestinal epithelial cells. Tryptophan metabolites produced by intestinal microflora during induction of colitis by bacterial infection in mice are known to play a protective role in the pathogenesis of the disease through AhR activation, which enhances IL-22 secretion (49, 50). Additionally, decreased tryptophan catabolism in the host alters the intestinal microflora and consequently alters the tryptophan metabolites produced by the microflora, thereby promoting differentiation of naive T cells into IL-22-producing cells (50).

## ACE2 and Tryptophan Metabolism in COVID-19

SARS-CoV2 targets host cells by binding of the receptor-binding domain of the spike protein expressed on its surface to angiotensin-converting enzyme 2 (ACE2) on the host cell surface. *ACE2* is located on the X chromosome and is expressed throughout the body, including the heart, blood vessels, lungs, kidneys, and brain, but predominantly in the small intestine (51–54). As suggested by its name, ACE2 was identified as the Angiotensin converting enzyme (ACE) homologue in renin-angiotensin system (RAS). It cleaves the vasoactive peptide angiotensin II to angiotensin 1-7 that has been reported to protect against the pathological effects of RAS activation in multiple organs (55, 56). Interestingly, ACE2 is involved in the absorption of neutral amino acids, particularly tryptophan, in the small intestine as a chaperone of the amino acid transporter, B0AT1 (57, 58). Indeed, reduced tryptophan absorption causes a decrease in brain serotonin (59) or impaired gut microbial ecology (57) in ACE2 knockout mice. Changes in intestinal microbiota in ACE2 knockout mice may also cause changes in tryptophan metabolites of intestinal bacteria, potentially affecting the regulation of immune responses by AhR activation (50).

Interestingly, several studies have reported that tryptophan metabolites regulate ACE2 expression *via* AhR (60, 61). Therefore, reduction in membrane-bound ACE2 potentially reduces risk of COVID-19. ACE2 is an organ-protective molecule that has been reported to protect against severe infectious diseases, including ARDS (62). In COVID-19, it is assumed that the expression of membrane-bound ACE2 is reduced by mechanisms such as internalization of SARS-CoV2 upon intracellular entry and cleavage to its soluble form by activation of TNF-α converting enzyme due to inflammation (63). Indeed, recent studies have suggested that the plasma concentration or activity of soluble ACE2 is associated with COVID-19 severity (64–66). Therefore, a vicious cycle is created by viral consumption of ACE2, which accelerates the development of COVID-19. Taken together, these results suggest that ACE2 involvement in tryptophan metabolism is specifically associated with COVID-19 pathogenesis in a complex manner (Figure 1B).

## Role of Tryptophan Metabolism and ACE2 in Aging

Tryptophan metabolism and activation of the kynurenine pathway are closely associated with aging (67). Blood tryptophan levels are reported to be lower in older individuals with frailty and cognitive impairment compared to healthy controls (68). Metabolites in the kynurenine pathway are also increased in subjects with frailty (69). Tryptophan metabolism is regarded as the *de novo* synthesis pathway of nicotinamide adenine dinucleotide (NAD^+^), which decreases in various organs with aging and is involved in the pathogenesis of diseases including obesity, diabetes, and Alzheimer's disease through energy metabolism and decreased sirtuin function (70). A recent study suggested that inhibition of an enzyme that negatively regulates this pathway enhances mitochondrial function and improves mammalian health (71). Deletion of ACE2 inhibits the promotion of aging phenotypes, including age-related muscle loss (sarcopenia) and skin atrophy, which is accompanied by enhanced *p16INK4a* (a senescence marker in mice) expression (72, 73). It is conceivable that the, overactivation of RAS does not contribute to the early aging phenotypes in ACE2 knockout mice. This is supported by the findings that both Mas (angiotensin 1-7 receptor) knockout and mice producing excessive angiotensin II by carrying both human renin and angiotensinogen genes did not exhibit the same aging phenotype as ACE2 knockout mice (74).

## Tryptophan Metabolism and Muscle Function

**Figure 2:**
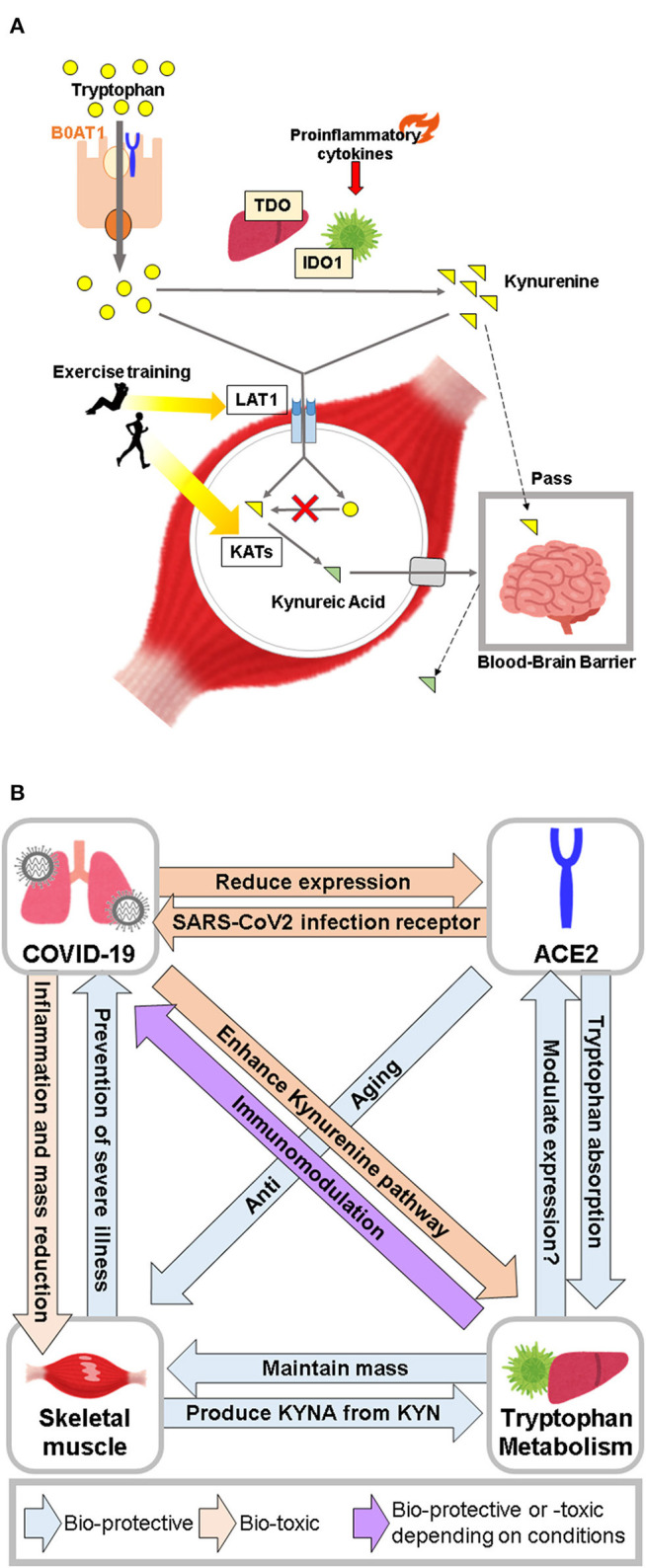
**(A)** Major role of skeletal muscle in tryptophan metabolism. Skeletal muscle takes up tryptophan and kynurenine, but does not metabolize kynurenine from tryptophan due to the absence of TDO and IDO. Skeletal muscle plays a neuroprotective role by converting kynurenine to kynurenic acid. **(B)** Conceptual diagram of the relationship between COVID-19, ACE2, tryptophan metabolism, and skeletal muscle.

Muscle protein anabolism is promoted by increased amino acid uptake in response to increased blood amino acid levels (75). Recent studies have shown that there is a significant difference in blood tryptophan concentration between frail and healthy elderly individuals. However, the results of metabolomic analysis in blood have been inconsistent (68, 76, 77), which could be attributed to several factors, including differences in the criteria used to select a frail study population, subject backgrounds, and experimental protocol details, including sample collection time, storage method, and measurement protocol. In contrast, metabolomic analysis of muscles has shown that frail elderly people have lower tryptophan levels than healthy elderly people, and exercise intervention enhances these levels (78). In this regard, the large neutral amino acid transporter 1, which is responsible for the uptake of tryptophan and kynurenine into skeletal muscle, has been shown to increase in response to acute resistance exercise in young and elderly individuals (79). Since expression of TDO and IDO is almost absent in skeletal muscle (80), kynurenine produced in the liver and other organs is considered to be the starting point of the kynurenine pathway in skeletal muscle. In contrast, kynurenine aminotransferase (KAT), the enzyme responsible for conversion of KYN to KYNA, is highly expressed in skeletal muscle (80), and its expression is increased by endurance training, resulting in increased circulating KYNA levels (81–83). KYN passes through the blood-brain barrier (BBB) and is involved in the pathogenesis of depression and schizophrenia. However, since KYNA does not pass through the BBB, the conversion of KYN to KYNA in skeletal muscle is thought to have neuroprotective effects (84). Studies in animal models have shown conflicting results on the effects of tryptophan and kynurenine on skeletal muscle. Administration of kynurenine to young mice induced oxidative stress along with increased lipid peroxides, and reduction in the size of muscle fibers (85). In contrast, the diameter of fibers in the tibialis anterior muscle of C57BL/6 mice fed a tryptophan-deficient diet was smaller than that of mice fed a standard diet, and blood myostatin (which promotes skeletal muscle breakdown) levels were increased (86). Thus elevated blood kynurenine levels may contribute to skeletal muscle atrophy, while an increase in blood tryptophan may contribute to skeletal muscle maintenance by inhibiting muscle degradation. The skeletal muscle damage observed in patients with COVID-19 may be due to various factors, such as the effect of cytokine storm or disuse due to inactivity (87). However, further investigation on the possible role of altered tryptophan metabolism in the pathogenesis of COVID-19 is warranted (Figure 2A).

## Perspectives

From a clinical perspective, it is important to investigate the utility of tryptophan supplementation for promotion of human health, particularly in the elderly, who are at high risk from COVID-19. Supplementation of tryptophan (a precursor of serotonin and melatonin) is widely used to improve mood and emotional functioning, and to treat insomnia (88). Studies in animal models have shown that a tryptophan-restricted diet is capable of delaying reproductive aging (89) and extending lifespan in female rats (90, 91). However, the effects of tryptophan depletion on quality of life at older ages remains unknown (92). Given the pro-aging phenotypes of ACE2 knockout mice independent of RAS, it is worth clarifying whether ACE2-mediated absorption of tryptophan provides protection from aging. In the case of inflammatory diseases, a high-tryptophan diet has been shown to reduce gut inflammation and severity of colitis (93). Conversely, perturbation of the intestinal microbiota also affects tryptophan metabolism. Probiotic supplements are capable of reducing upper respiratory tract infections accompanied by the attenuation of exercise-induced tryptophan degradation rates in trained athletes (94). Furthermore, AhR activation by tryptophan metabolites can increase or decrease ACE2 expression as mentioned earlier. In humans as well as mice, tryptophan supplementation has been found to elevate plasma levels of tryptophan metabolites, KYN, and KYNA (95) therefore, it is clinically relevant to understand the role of tryptophan supplementation on organ ACE2 levels, and its relation to COVID-19 susceptibility.

## Conclusion

In patients with severe COVID-19, activation of the kynurenine pathway by various cytokines, and tryptophan metabolites regulate immune responses through AhR and GPR35 activation. In contrast, ACE2, which is the primary receptor for SARS-CoV2 infection and has an anti-aging function, is involved in absorption of tryptophan from the small intestine. Furthermore, tryptophan metabolites regulate expression of ACE2 *via* AhR. These data suggest that ACE2 serves as a gateway to SARS-CoV2 infection, and may also play an immunomodulatory role in the pathogenesis of COVID-19 through mutual regulation of tryptophan metabolites. Furthermore, skeletal muscle may contribute to immunoregulatory mechanisms mediated *via* tryptophan metabolites by storing tryptophan and producing KYNA (Figure 2B).Collectively, tryptophan metabolism, ACE2, and skeletal muscle are closely linked to the severity of COVID-19. Further studies are needed to elucidate the role of tryptophan supplementation in the maintenance of human health in relation to its effect on ACE2 and skeletal muscle in the COVID-19 era.

## Author Contributions

HT and KY were equally responsible for conceptualization, drafting, writing, and editing. All authors contributed to the article and approved the submitted version.

## Conflict of Interest

The authors declare that the research was conducted in the absence of any commercial or financial relationships that could be construed as a potential conflict of interest.

## Publisher's Note

All claims expressed in this article are solely those of the authors and do not necessarily represent those of their affiliated organizations, or those of the publisher, the editors and the reviewers. Any product that may be evaluated in this article, or claim that may be made by its manufacturer, is not guaranteed or endorsed by the publisher.

## Abbreviations

ACE2: angiotensin converting enzyme 2
ARDS: acute respiratory distress syndrome
AhR: aryl hydrocarbon receptor
BBB: blood-brain barrier
COVID-19: coronavirus disease 2019
FICZ: 6-formylindolo[3,2-b]carbazole
GPR35: G protein-coupled receptor 35
IDO: indoleamine-2, 3-dioxygenase
KA:K: KYNA to KYN
KAT: kynurenine aminotransferase
KYN: kynurenine
KYNA: kynurenic acid
NAD^+^: nicotinamide adenine dinucleotide
RAS: renin-angiotensin system
SARS-CoV2: severe acute respiratory syndrome coronavirus 2
TDO: tryptophan-2, 3-dioxygenase
Treg: regulatory T.
